# *In vivo* neuroimaging evidence of hypothalamic alteration in Prader–Willi syndrome

**DOI:** 10.1093/braincomms/fcac229

**Published:** 2022-09-09

**Authors:** Stephanie S G Brown, Katherine E Manning, Paul Fletcher, Anthony Holland

**Affiliations:** Department of Psychiatry, University of Cambridge, Addenbrookes Hospital, Cambridge CB2 0QQ, UK; Department of Psychiatry, University of Cambridge, Addenbrookes Hospital, Cambridge CB2 0QQ, UK; Department of Psychiatry, University of Cambridge, Addenbrookes Hospital, Cambridge CB2 0QQ, UK; Department of Psychiatry, University of Cambridge, Addenbrookes Hospital, Cambridge CB2 0QQ, UK

**Keywords:** hypothalamus, Prader–Willi, structural MRI, obesity, hyperphagia

## Abstract

Prader–Willi syndrome is a genetic neurodevelopmental disorder with an early phenotype characterized by neonatal hypotonia, failure to thrive, and immature genitalia. The onset of hyperphagia in childhood and developmental, physical and neuropsychiatric characteristics indicate atypical brain development and specifically hypothalamic dysfunction. Whether the latter is a consequence of disruption of hypothalamic pathways for genetic reasons or due to a failure of hypothalamic development remains uncertain. Twenty participants with Prader–Willi syndrome, 40 age-matched controls and 42 obese participants underwent structural MRI scanning. The whole hypothalamus and its subnuclei were segmented from structural acquisitions. The Food-Related Problem Questionnaire was used to provide information relating to eating behaviour. All hypothalamic nuclei were significantly smaller in the Prader–Willi group, compared with age and gender matched controls (*P* < 0.01) with the exception of the right anterior–inferior nucleus (*P* = 0.07). Lower whole hypothalamus volume was significantly associated with higher body mass index in Prader–Willi syndrome (*P* < 0.05). Increased preoccupation with food was associated with lower volumes of the bilateral posterior nuclei and left tubular superior nucleus. The whole hypothalamus and all constituent nuclei were also smaller in Prader–Willi syndrome compared with obese participants (*P* < 0.001). Connectivity profiles of the hypothalamus revealed that fractional anisotropy was associated with impaired satiety in Prader–Willi syndrome (*P* < 0.05). We establish that hypothalamic structure is significantly altered in Prader–Willi syndrome, demonstrating that hypothalamic dysfunction linked to eating behaviour is likely neurodevelopmental in nature and furthermore, distinctive compared with obesity in the general population.

## Teaser

Prader–Willi syndrome is a neurodevelopmental disorder with a phenotype that includes hyperphagia, which can lead to life-threatening obesity. Brown *et al.* report for the first time *in vivo* neuroimaging evidence of reduced volumes of the hypothalamus in young adults with Prader–Willi syndrome, with a significant relationship to increased food-related problems.

## Graphical Abstract

**Graphical abstract:**
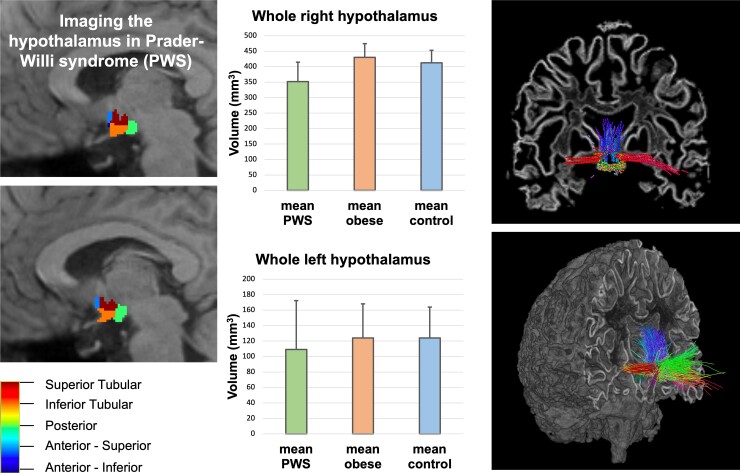

## Introduction

Prader–Willi syndrome (PWS) is a neurodevelopmental disorder caused by the absence of paternal expression of maternally imprinted genes at the chromosomal locus 15q11-13. Phenotypically, PWS is characterised by developmental delay, intellectual disability, relative sex and growth hormone deficiencies of hypothalamic origin, dysregulation of emotions and severe hyperphagia that can cause life-threatening obesity. Hyperphagia and obesity are the main cause of metabolic complications and increased morbidity and mortality, however, the biological mechanisms and the reasons for dysfunctional hypothalamic pathways responsible for this phenotype are largely unknown.^1^ Studies^2,3^ indicate that satiety responses to food intake are significantly delayed and transitory in PWS. It has been demonstrated that PWS is associated with a greatly increased threshold for satiety, reporting reduced or absent satiety responses after large calorie intake,^4^ and limbic and para-limbic regions, which are thought to motivate eating behaviour, remain hyperactive in PWS after eating, suggesting impairment of the satiety response.^5^

The hypothalamus and associated projections regulate food intake and satiety perception, and evidence from a host of animal studies has shown that lesions of the ventromedial hypothalamic nuclei result in hyperphagia and evoke weight gain.^6–8^ Dysregulation of the endocrine satiety signals directly and indirectly arising from the hypothalamic neural circuitry are well-known in PWS.^9,10^ Major advances in our understanding of hypothalamic pathways involved in appetite have elucidated core neuroendocrine mechanisms whose disruption may account for features of PWS.^11^ For example, obestatin, acting on the arcuate nucleus of the hypothalamus has been shown to contribute to poor feeding in early years of PWS.^12,13^ Leptin similarly acts on the arcuate nucleus of the hypothalamus to stimulate pro-opiomelanocortin and inhibit neuropeptide Y, leading to melanocortin receptor-mediated induction of satiety. Abnormal leptin signalling in PWS is linked to hyperphagia and non-insulin dependent diabetes.^14^ Neuroanatomical tracing techniques have demonstrated that the paraventricular nucleus, located in the superior tubular region of the hypothalamus, is integral to gustatory neural circuitry, with major connections to the dorsal vagal complex, the nucleus of the solitary tract and the parabrachial area.^6^ Disruption of this circuitry due to single gene mutations, polygenic factors,^15^ or for developmental or structural reasons all impact on appetite control and risk of obesity; understanding which is relevant to PWS may have significant therapeutic relevance.

Due to the small size of the hypothalamus and lack of defined image contrast in neuroimaging acquisitions, studies examining its structure and function in humans *in vivo* are scarce.^16–19^ Here, we apply a recently developed automated tool to segment the hypothalamus in PWS for the first time,^20^ to test our hypothesis that disordered hypothalamic development plays a critical role in the dysregulation of eating behaviour in PWS.

## Methods and materials

### Participants

Young adults with PWS had been recruited from across England for an earlier neuroimaging study.^21^ The inclusion criteria had been: aged between 18 and 28 years inclusive, genetic confirmation of PWS diagnosis, capacity to consent and ability to comply with MRI assessment. Exclusion criteria were contraindication for MRI scanning or inability to tolerate MRI environment, current psychiatric disorder which would disrupt ability to take part in the study, current or past history of neurological disorders or trauma (including epilepsy, head injury or loss of consciousness) and current or recent (within 12 months) participation in a clinical trial of an investigational medicinal product. Control participants for the earlier study had been recruited from the NeuroScience in Psychiatry Network U-Change project. Inclusion criteria included: willingness and ability to give informed consent and aged 14–28 years inclusive. Control exclusion criteria was contraindication for MRI, currently being treated for a psychiatric disorder, alcohol or drug dependence, current or past history of neurological disorder or trauma, current or recent participation in a clinical trial, learning disability requiring specialist educational support and/or medical treatment and inability to understand written or spoken English. Obese control participants inclusion and exclusion criteria was the same as for the control criteria, with the addition of having a body mass index (BMI) of over 25 for inclusion. This group had previously been part of neuroimaging studies.^22^

### Behavioural, neuropsychological and clinical data collection

Data regarding basic personal information, height, weight and BMI were recorded for each participant. Participants with PWS completed assessments of intelligence quotient (IQ) using the complete Wechsler Adults Intelligence Scales Fourth Edition,^23^ and control participant IQ was assessed using a shortened version of the WAIS, including the vocabulary and matrix reasoning subsections of the Wechsler Abbreviated Scale of Intelligence Second Edition.^24^ PWS participants also had genetic testing for genetic subtype of PWS and completed the Food-Related Problem Questionnaire (FRPQ),^25^ which is a measure of eating behaviour designed for use in the PWS population. Subscales of the FRPQ include: preoccupation with food, satiety impairment and other negative food-related behaviour. The FRPQ is a 16-item questionnaire, the maximum score for which is 96, and the maximum scores for subscales are as follows: preoccupation with food, 18; impairment of satiety, 30; composite negative behaviour, 48. As described by Russell and Oliver^25^, the preoccupation with food subscale refers to hypervigilance with regard to food and excessive references to food, the impairment of satiety subscale refers to any indication that the person is not satiated and the composite negative behaviour subscale refers to inappropriate behaviour with regard to food access or storage.

### MRI acquisition

All participants underwent MRI scanning using a Siemens MAGNETON 3 T Trio system, using a 32-channel head coil. The T_1_ scans were acquired with a repetition time (TR) of 18.7 ms, flip angle was 20 degrees, field of view was 256 × 256 mm, with a slice thickness of 1 mm, voxel size of 1 mm × 1 mm and a total of 176 slices. Diffusion scans were acquired using a two-dimensional echo-planar sequence with a TR of 8.7 ms, flip angle of 90 degrees and a slice thickness of 2 mm.

### MRI data analysis

**Figure 1:**
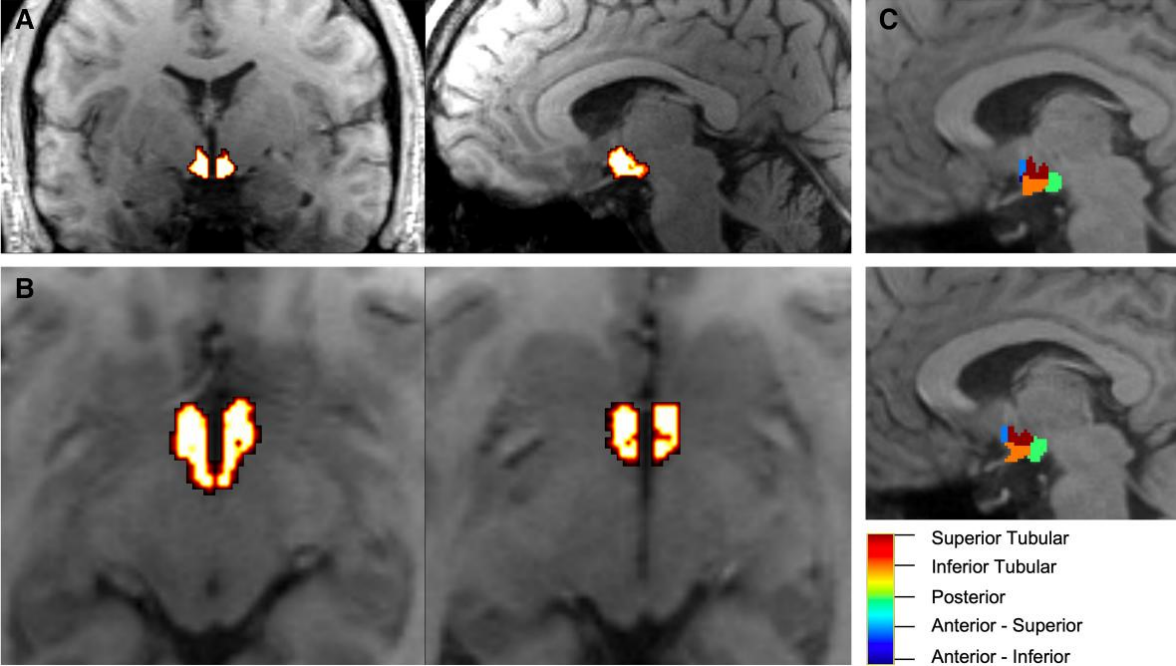
**Hypothalamic segmentation.** (**A**) Coronal and sagittal slices of the whole hypothalamic segmentation overlaid on the T_1_-weighted image. (**B**) Two axial slices of the whole hypothalamic segmentation, overlaid on the T_1_-weighted image. (**C**) Individual hypothalamic nuclei segmentations.

T_1_-weighted acquisitions were pre-processed using the FreeSurfer v7.0 ‘recon-all’ pipeline (https://surfer.nmr.mgh.harvard.edu/fswiki/recon-all). Pre-processed structural data were then segmented to extract the whole hypothalamus and hypothalamic nuclei using an automated tool based on a deep convolutional neural network in FreeSurfer development version 7^20^ (Fig. 1). Subnuclei per hemisphere were segmented and labelled as follows: anterior–inferior, anterior–superior, posterior, tubular inferior and tubular superior. Hypothalamic volume data were also normalized to intracranial volume (ICV).^26^ For all participant data, acquisitions segmentations were manually inspected for quality assurance. Visual inspection was carried out by using overlay onto the T_1_-weighted images and inspecting good quality alignment to neuroanatomy.

Diffusion-weighted acquisitions were denoised using MRTrix3.^27^ B_1_ field inhomogeneity correction was applied to the diffusion images using the MRTrix3 command ‘dwibiascorrect’,^27^ and the fibre orientation distribution (FODs) images were created using constrained super-resolved spherical deconvolution.^28,29^

Segmented hypothalamic nuclei were co-registered to diffusion space (B0 image) using Statistical Parametric Mapping software (SPM12) with nearest neighbour interpolation. The MRTrix fsl command ‘5ttgen’ was used to produce a five tissue-type segmentation image, which was then used the create a mask of the grey matter–white matter interface. Tractograms of the hypothalamic white matter cortical connectivity were created using 1000 seeds per voxel within the hypothalamic mask, with default parameters of 0.1 mm × voxel size for individual step size for probabilistic building of streamlines. As altered cortical–hypothalamic connectivity in PWS was hypothesised, all selected streamlines were constrained to connectivity with the grey–white matter interface mask, which also included the subcortical grey matter. FOD amplitude cut-off was 0.12 and the maximum angle between successive steps was 90° × step size × voxel size. Spherical deconvolution informed filtering of the tractograms was then carried out (‘SIFT2’)^30^ to remove streamlines unlikely to be relevant to underlying ground truth anatomy. Streamline density was extracted from the tractogram data, along with means of fractional anisotropy (FA), mean diffusivity, axial diffusivity and radial diffusivity of the streamlines.

### Statistical analysis

A power calculation was performed to determine adequate sample size to reach at least 80% power for statistical analyses. Between group comparisons were performed using parametric *t*-testing and association analyses were performed using linear model regression. All statistical analysis was carried out in R version 3.6.1.

### Data availability

Data will be made available upon reasonable request.

## Results

### Participant demographics

Twenty participants with genetic confirmation of PWS (14 female, 6 male), forty neurotypical age- and sex matched controls (26 female, 14 male) and forty-two obese participants (24 female, 18 male), who had previously undergone a 3 T MRI scan acquisition, were included. Obese participants were matched for BMI to the PWS group. All participants successfully completed assessment for IQ, and 19 of the 20 PWS participants completed the FRPQ. As expected, the PWS group had significantly lower IQ and significantly higher BMI than the non-obese control group. Participant demographics are summarised in Table 1. Nineteen of the 20 PWS participants had the deletion subtype of PWS, and one had the maternal uniparental disomy (mUPD) subtype. The participant with mUPD was not atypical of the cohort in regards to measures of behaviour, IQ or brain structure.

**Table 1:** Participant demographics

	PWS mean	PWS SD	Control mean	Control SD	Obese mean	Obese SD	PWS-control *P*-value	PWS-obese *P*-value
Age (years)	23.1	2.3	22.8	2.2	30.4	6.1	**0.73**	***
IQ	63.1	11.9	112.9	11.3			***	
BMI	30.8	7.2	24.1	3.8	31.0	5.0	***	**0.56**
FRPQ Preoccupation	12.3	3.9						
FRPQ Impaired satiety	22.6	4.4						
FRPQ negative behaviour	25.7	10.3						
FRPQ total	62.2	14.6						

Means, standard deviations (SD) and *t*-test *P*-value results of participant demographics for PWS, non-obese and obese control groups (* = *P* < 0.05, ** = *P* < 0.01, *** = *P* < 0.001).

IQ, intelligence quotient; BMI, body mass index; FRPQ, Food-Related Problems Questionnaire.

### Hypothalamic volumetrics

**Figure 2:**
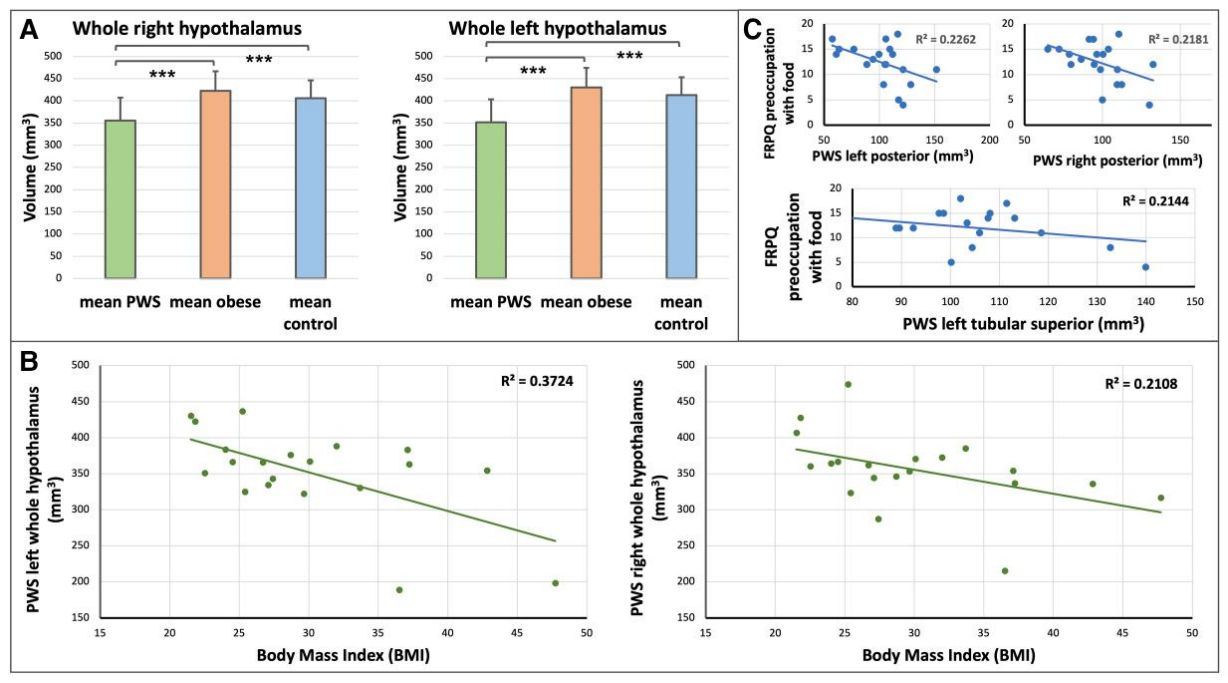
**Volumetric analysis of the hypothalamus.** (**A**) Mean whole hypothalamic volumes for the PWS, obese and non-obese control groups (Parametric *t*-testing results, PWS *n* = 20, Controls *n* = 40, Obese *n* = 42; *** = *P* < 0.001). (**B**) BMI measurements are significantly associated with the left and right hypothalamic volumes in the PWS group (left: *P* < 0.05, *R*^2^ = 0.3724, right: *P* < 0.05, *R*^2^ = 0.2108). (**C**) Food-Related Problem Questionnaire (FRPQ) measures of preoccupation with food are significantly associated with volume of the bilateral posterior and left tubular superior hypothalamic nuclei in PWS (left posterior: *P* < 0.05, *R*^2^ = 0.2262, right posterior: *P* < 0.05, *R*^2^ = 0.2181, left tubular superior: *P* < 0.05, *R*^2^ = 0.2144).

All participants showed visually good quality neuroanatomical alignment of segmentation. Volumetric data results for the hypothalamus and constituent nuclei for all participants is summarized in Table 2, and normalized to ICV data is presented in Table 3. All left hemisphere hypothalamic nuclei and all right hemisphere hypothalamic nuclei except the anterior–inferior nucleus were significantly smaller in PWS compared with the control group for both the normalized and non-normalized to ICV data. Both the left and right whole hypothalamus was markedly significantly smaller in PWS compared with controls (Fig. 2A). Lower whole hypothalamus volume was also significantly associated with increased BMI in PWS (Fig. 2B), but hypothalamic volume did not show an association with IQ or age. Furthermore, the left posterior, left tubular superior and right posterior nuclei displayed a significant negative association with preoccupation with food as measured by the FRPQ (Fig. 2C), with the aforementioned left nuclei volumes also exhibiting a similar negative association with BMI. After normalisation to ICV, the left tubular superior nucleus only exhibited a significant negative association with both preoccupation with food and BMI. Results of all associative analyses within the PWS group for both normalized and non-normalized to ICV data are presented in Supplementary Fig. 1. These relationships demonstrate a scalable association of smaller hypothalamic nuclei and increasing levels of food preoccupation, and it is expected outcome of increased weight.

**Table 2:** Neuroimaging between group results

	PWS mean	PWS SD	Control mean	Control SD	Obese mean	Obese SD	PWS-control *P*-value	PWS-obese *P*-value
Hypothalamic nuclei volumes (mm^3^)								
Left anterior–inferior	15.98	4.43	18.79	3.87	19.12	4.51	*	**
Left anterior–superior	20.89	4.67	26.28	4.35	24.84	4.51	***	**
Left posterior	101.84	23.63	122.71	16.78	116.95	17.46	***	**
Left tubular inferior	111.97	20.88	131.31	14.66	149.40	18.03	***	***
Left tubular superior	100.87	22.25	113.72	14.72	119.72	13.93	**	***
Left whole	351.57	63.04	412.83	39.92	430.05	44.25	***	***
Right anterior–inferior	14.10	5.01	15.92	2.92	16.75	3.78	**0.07**	*
Right anterior–superior	21.98	5.99	26.37	4.16	25.32	4.86	**	*
Right posterior	98.12	17.11	113.58	15.66	120.41	17.04	***	***
Right tubular inferior	111.71	17.97	125.82	13.88	135.77	16.59	**	***
Right tubular superior	109.25	23.16	123.97	17.02	123.98	14.55	**	**
Right whole	355.57	51.95	405.67	40.13	422.24	44.05	***	***
Hypothalamic–cortical connectivity								
Connection density	3795.6	2514.75	3849.3	1933.1			**0.93**	
Axial diffusivity	0.00152	0.00012	0.00149	0.00022			**0.61**	
Fractional anisotropy	0.32381	0.02712	0.32381	0.08256			**0.94**	
Mean diffusivity	0.00115	0.00008	0.00114	0.00022			**0.94**	
Radial diffusivity	0.00096	0.00007	0.00096	0.00023			**0.89**	

Means, standard deviations (SD) and *t*-test *P*-value results of structural neuroimaging data (* = *P* < 0.05, ** = *P* < 0.01, *** = *P* < 0.001).

**Table 3:** Hypothalamic volume between group results normalized to intracranial volume (percentage value)

	PWS mean	PWS SD	Control mean	Control SD	Obese mean	Obese SD	PWS-control *P*-value	PWS-obese *P*-value
Hypothalamic nuclei volumes								
Left anterior–inferior	0.0010	0.0003	0.0012	0.0003	0.0013	0.0002	*	***
Left anterior–superior	0.0014	0.0003	0.0017	0.0004	0.0017	0.0003	***	***
Left posterior	0.0066	0.0016	0.0082	0.0018	0.0079	0.0008	**	***
Left tubular inferior	0.0072	0.0016	0.0088	0.0022	0.0102	0.0009	**	***
Left tubular superior	0.0065	0.0015	0.0075	0.0016	0.0081	0.0007	*	***
Left whole	0.0228	0.0048	0.0270	0.0058	0.0292	0.0018	**	***
Right anterior–inferior	0.0009	0.0003	0.0010	0.0004	0.0011	0.0002	**0.08**	**
Right anterior–superior	0.0014	0.0004	0.0017	0.0005	0.0017	0.0003	**	***
Right posterior	0.0063	0.0013	0.0075	0.0017	0.0082	0.0010	**	***
Right tubular inferior	0.0071	0.0012	0.0084	0.0017	0.0092	0.0009	*	***
Right tubular superior	0.0070	0.0015	0.0082	0.0021	0.0084	0.0007	**	***
Right whole	0.0228	0.0039	0.0270	0.0058	0.0287	0.0020	**	***

Means, standard deviations (SD) and *t*-test *P*-value results of hypothalamic volumes as normalized* to intracranial volume (ICV) (*hypothalamic volume as a percentage of ICV); (* = *P* < 0.05, ** = *P* < 0.01, *** = *P* < 0.001).

To examine whether this relationship between a reduced hypothalamic volume and BMI was specific to PWS and also whether this relationship might be driven by obesity irrespective of the cause, we compared the hypothalamic structures in PWS to an obese control group. We found that all measures of hypothalamic volume were significantly smaller bilaterally in PWS compared with the obese control group (Fig. 2A). Whole hypothalamic volume did not differ between the general population obese and non-obese controls, and moreover, unlike in PWS, obese control participants did not show a relationship between hypothalamic nuclei volumes and BMI. Results of all associative analyses within the obese group for both normalized and non-normalized to ICV data are presented in Supplementary Fig. 2.

### Hypothalamic structural connectivity

**Figure 3:**
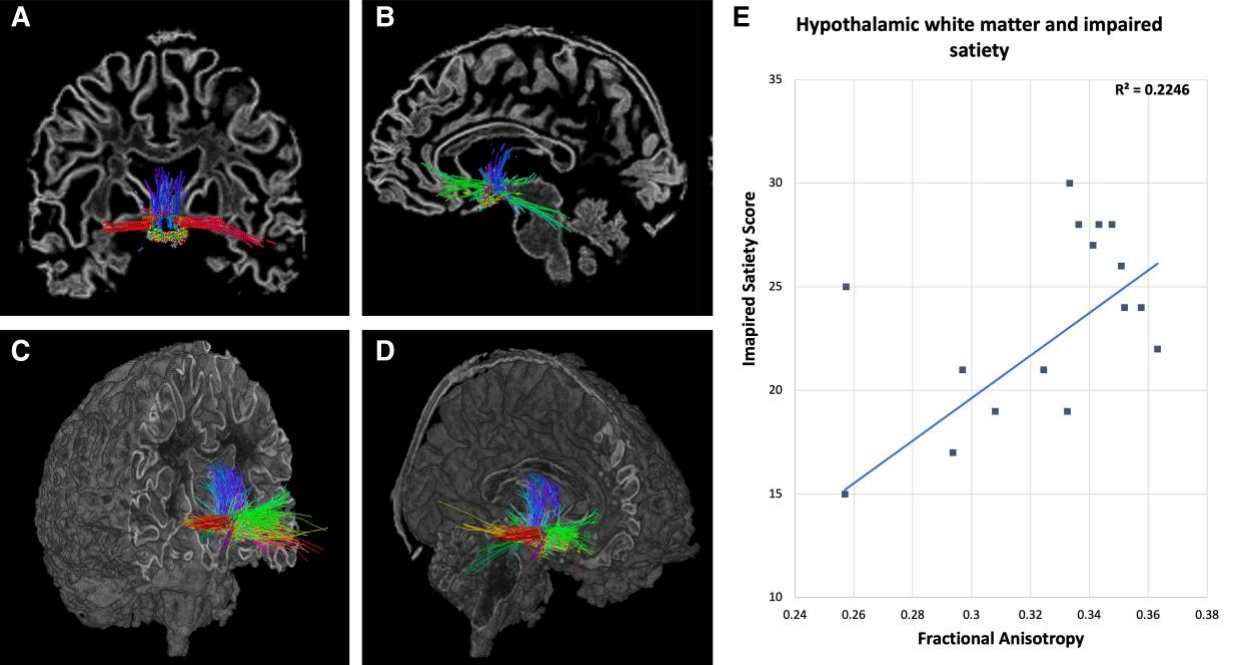
**Hypothalamic white matter connectivity.** (**A**) Coronal brain slice showing tractography seeded in the hypothalamus. (**B**) Sagittal brain slice showing tractography seeded in the hypothalamus. (**C** and **D**) Coronal and sagittal sliced brain volumes showing the hypothalamic connectivity profile extending rostrally to the basal forebrain and prefrontal cortical areas, caudally to the cerebellar cortex and close-range connectivity with the thalamus and upper brainstem. Colours represent directionality of tracts. (E) Impaired satiety score exhibts a positive correlative relationshipwith fractional anisotropy derived from hypothalamic sturctural connectivity.

Seventeen PWS participants and 37 control participants were included in an analysis of hypothalamic white matter connectivity, with three PWS and three control participants from the full data set excluded due to insufficient diffusion data quality. Tractograms created from seeds within the hypothalamic structure exhibit a connectivity profile of rostral projections to the basal forebrain and prefrontal areas, caudal projections via the fornix and terminal stria, cerebellar connectivity, close-range thalamic connectivity and upper brainstem connectivity (Fig. 3A–D). Structural connectivity data of the hypothalamus for all participants is summarised in Table 2. Measures of hypothalamic white matter connectivity to the cortex did not show any significant alteration in individuals with PWS compared with controls. However, correlation plotting of microstructural brain measures revealed that increased FA of the hypothalamic connectivity profile was associated with greater levels of impaired satiety (*P* = 0.03) (Fig. 3E), suggesting that in PWS, strengthening of hypothalamic–cortical connectivity impairs the satiety response.

## Discussion

The structure and white matter connectivity of the hypothalamus in PWS have not previously been investigated *in vivo*, and therefore these data are the first to present evidence in humans with PWS of altered hypothalamic anatomy. Our primary finding of a significant reduction in size of the hypothalamus and constituent nuclei in PWS compared to controls, and the relationship with BMI and preoccupation with food as measured by the FRPQ, is strongly suggestive of dysregulation of hypothalamic control of appetite in PWS. Moreover, we establish that in an obesity group without neurodevelopmental disorder, matched for BMI to the PWS group, hypothalamic volume was equivalent to healthy controls. We therefore show that abnormal structure and volume of the hypothalamus is neither a general consequence of obesity nor is it present in other causes of obesity. In PWS, hypothalamic dysfunction and the associated impairments in the regulation of energy intake and in growth and sex hormone regulation is neurodevelopmental in nature. Further to this, we posit that early, potentially pre-natal, development of the hypothalamic structures is impaired in PWS, and may contribute to the early hypophagia leading later to hyperphagia. Concerning hypothalamic–cortical white matter connectivity, we also establish a significant association of pathway microstructural order and impaired satiety as measured by the FRPQ, evidencing that enhancement of the hypothalamic–cortical circuitry impairs the satiety reflex in PWS.

Marked reductions in hypothalamic volume in PWS, especially in the context of no significant relationship with age in this sample of young adults, potentially indicates a developmental deficit of hypothalamic growth. We postulate that overall reduction in size of the hypothalamus and constituent nuclei in PWS results in reduced receptor availability for anorexigenic signalling and dysregulation of food-related reward processing. Our results show that reduced posterior hypothalamus volumes correlate with increased preoccupation with food, which highlights the importance of the hypothalamus in reward circuitry.^31^ We find that the posterior nuclei, which contain the mamillary bodies, exhibit one of the most marked reductions in size in PWS. Interestingly, the mamillary bodies facilitate reward and goal-directed behaviour via reciprocal connections to the ventral tegmental area,^32^ suggesting that in PWS, a significant underlying mechanism for weight gain is through a lack of suppression of reward-related food preoccupation following food intake. The inferior tubular hypothalamic region also shows a highly significant reduction in size in PWS compared to both obese and non-obese controls. The inferior tubular area contains the arcuate and infundibular regions, which are sites of leptin-induced inhibition of neuropeptide Y and anorexigenic signalling, inducing satiety through melanocortin receptor activity.^14^ Our finding of significant reduction in size of the infundibular and arcuate-containing area of the hypothalamus supports the leptin-resistance hypothesis of PWS, with our results likely highlighting an underlying causative mechanism of lack of receptor availability due to decreased grey matter volume.

Previous investigations into white matter structural connectivity in PWS have shown that prefrontal cortex, anterior cingulate and temporal lobe connections exhibited reduced FA.^33^ Our findings concerning hypothalamic–cortical connectivity, specifically in PWS, show that similar significant reductions of FA or other measures of microstructural order are not evident, but instead a graded relationship exists between impaired satiety and FA. This suggests that, while connection density and integrity of hypothalamic structural connectivity is comparable to healthy controls, white matter microstructure indicative of alterations to axonal thickness or myelination is closely linked with an impaired satiety reflex in PWS. This finding aligns with previous functional MRI research in PWS, with heightened activations in the hypothalamic region post-meal indicating that the hypothalamus is hyperactive during times when satiety signals would be expected.^34^ Maps of probabilistic tractography seeded at the hypothalamus show that white matter tracts projecting to the prefrontal cortex are a key connection.^35^ The prefrontal cortices are often associated with reward in the context of eating behaviours, and functional neuroimaging shows that greater BOLD response in the prefrontal cortices is associated with larger food intakes,^36^ supporting the hypothesis that the continuation of eating is at least partially underpinned by a surfeit of reward processing. Moreover, recent findings show that the deep cerebellar nuclei terminate food intake via increases in striatal dopamine and that in groups with a genetic disorder characterised by insatiable appetite, cerebellar response to food is markedly different.^37^ With the evidenced structural connectivity between the hypothalamus and cerebellum, our results indicate a role for this pathway in poor appetite control in PWS.

As highly significant volume differences between the PWS and the obese control group were identified across all nuclei regions and hypothalamic volume did not show any association with age in PWS, it is likely that reduced hypothalamic size in this young adult population is a stable characteristic that is potentially developmental in nature and not attributable to the complex interacting effects of increased BMI. An increased mean age of 7 years in the BMI-matched obesity group compared with the PWS group may have however, despite lack of an association with age, had a statistically small effect on these results. We also show that the hypothalamic connective profile in PWS is associated with behaviourally diminished satiety, together suggesting that these mechanistic underpinnings of overeating may be considered a promising future therapeutic target. PWS is due to the absence of expression of maternally imprinted gene(s),^38^ a class of genes known to drive placental–foetal nutritional pathways.^39^ Intranasal oxytocin is being tried experimentally to treat various aspects of the early and later PWS phenotypes, including the hyperphagia, and growth and sex hormone replacement therapy is now routine, both seeking to compensate for the deficits of hypothalamic origin. However, this study raises a more fundamental question of potential therapeutic significance: Why is the development of the hypothalamus impaired in PWS? We propose that this may either be a direct effect of the absence of foetal expression of maternally imprinted genes or, alternative, an indirect effect through the down-regulation of placental–foetal nutritional pathways consequent upon the foetal genotype.^40^ The resultant exposure of the PWS foetus to a restricted nutritional environment we suggest may impact on subsequent leptin-induced foetal hypothalamic development pre- and post-natally, which has been shown to be dependent on nutritional status.^41,42^ These hypotheses require further study in animal models of PWS and where possible longitudinally in humans, and here we present preliminary evidence in young adults of hypothalamic dysfunction.

## Supplementary Material

fcac229_Supplementary_Data

## Abbreviations

BMI =: body mass index
FOD =: fibre orientation distribution
FRPQ =: Food-Related Problem Questionnaire
FA =: fractional anisotropy
IQ =: intelligence quotient
mUPD =: maternal uniparental disomy
PWS =: Prader–Willi syndrome
TR =: repetition time
