# Supplementary material for: In vivo neuroimaging evidence of hypothalamic alteration in Prader–Willi syndrome
Source: Brain Commun. 2022 Sep 9;4(5):fcac229. doi: 10.1093/braincomms/fcac229 (PMC9487704; doi:10.1093/braincomms/fcac229)
Supplement: fcac229_Supplementary_Data [file fcac229_supplementary_data.docx]

**
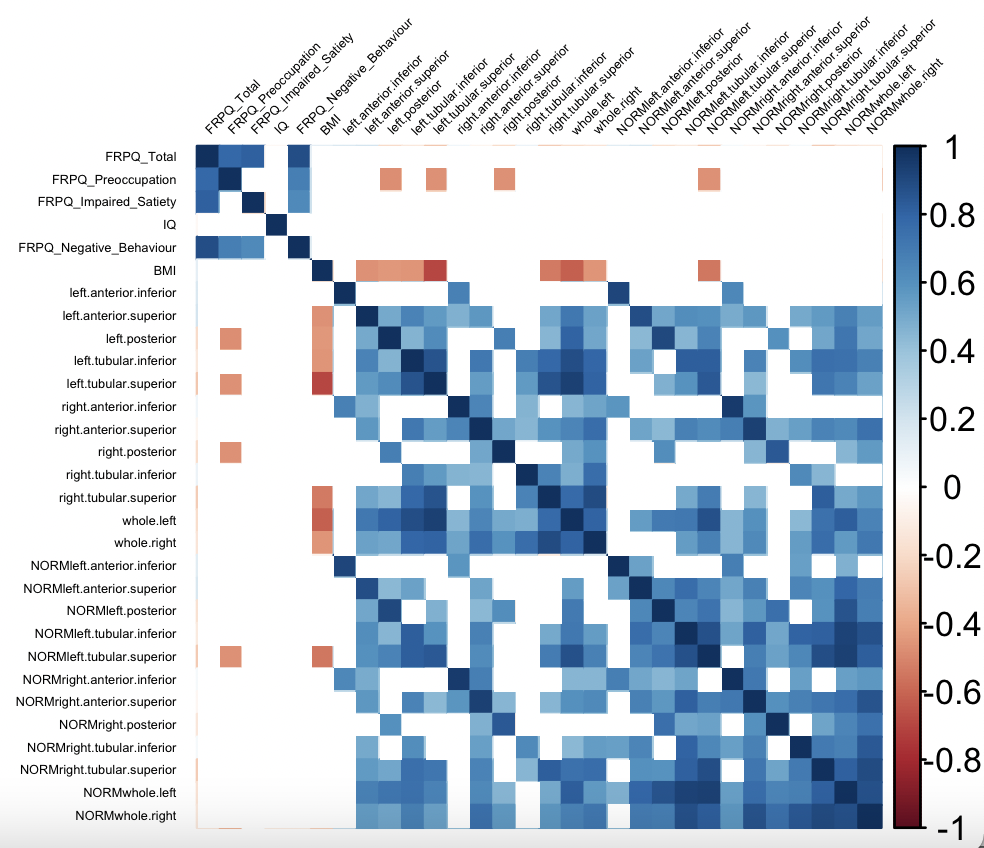
**

**Supplementary Fig. 1** Correlation plot of within PWS group associative analyses of hypothalamic volumes both normalised (NORM) and non-normalised to ICV, with blue-red colour bar representing correlation coefficients and non-significant associations represented by white.

**
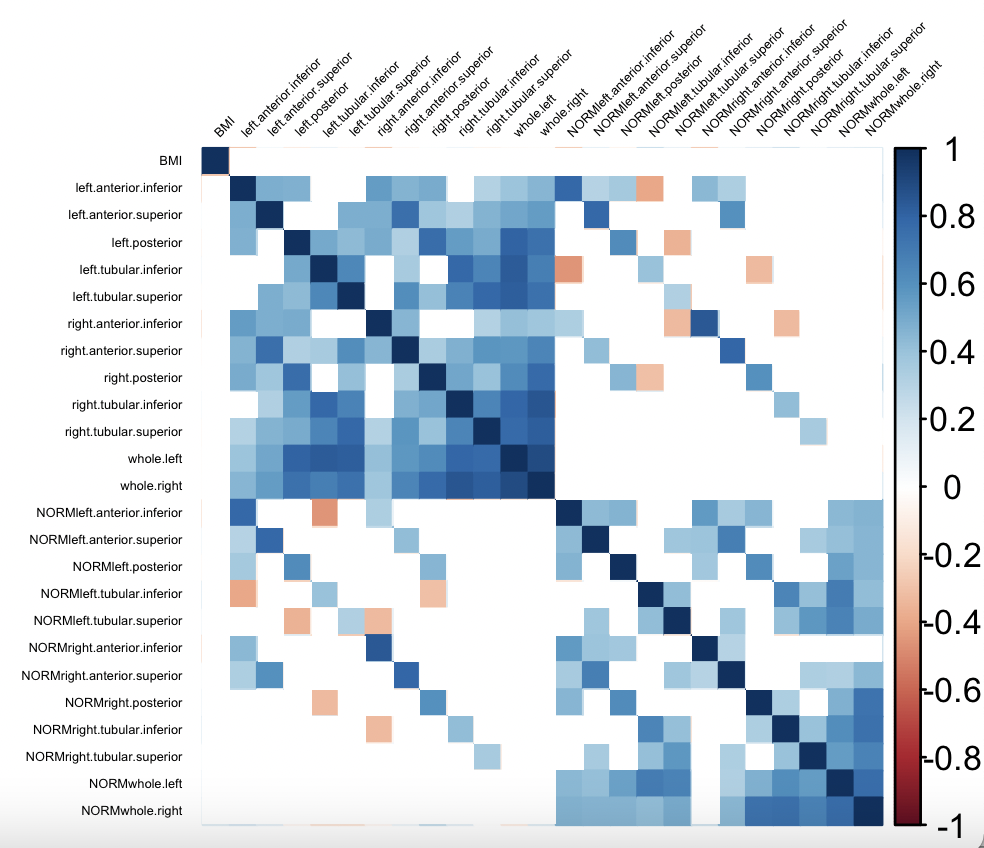
**

**Supplementary Fig. 2** Correlation plot of within obese group associative analyses of hypothalamic volumes both normalised (NORM) and non-normalised to ICV, with blue-red colour bar representing correlation coefficients and non-significant associations represented by white.
